# Reds are more important than greens: how UK supermarket shoppers use the different information on a traffic light nutrition label in a choice experiment

**DOI:** 10.1186/s12966-015-0319-9

**Published:** 2015-12-12

**Authors:** Peter Scarborough, Anne Matthews, Helen Eyles, Asha Kaur, Charo Hodgkins, Monique M Raats, Mike Rayner

**Affiliations:** British Heart Foundation Centre on Population Approaches for Non-Communicable Disease Prevention, Nuffield Department of Population Health, University of Oxford, Oxford, UK; National Institute for Health Innovation and Department of Epidemiology and Biostatistics, The University of Auckland, Auckland, New Zealand; Food, Consumer Behaviour and Health Research Centre, University of Surrey, Guildford, UK

**Keywords:** Traffic light, Nutrition label, Food label, Front of pack nutrition labelling, Colour coded nutrition labelling

## Abstract

**Background:**

Colour coded front-of-pack nutrition labelling (‘traffic light labelling’) has been recommended for use in the UK since 2006. The voluntary scheme is used by all the major retailers and some manufacturers. It is not clear how consumers use these labels to make a single decision about the relative healthiness of foods. Our research questions were: Which of the four nutrients on UK traffic light labels (total fat, saturated fat, sugar and salt) has the most influence on decisions? Do green lights or red lights have a greater influence? Are there age and gender differences in how people use the colour and nutrient information?

**Methods:**

We recruited participants from a UK supermarket chain membership list to conduct an online choice experiment in May 2014. We analysed data using multilevel logisitic models with food choices (*n* = 3321) nested in individuals (*n* = 187) as the unit of analysis.

**Results:**

A food with more reds was 11.4 (95 % confidence intervals: 10.3, 12.5) times less likely to be chosen as healthy, whereas a food with more greens was 6.1 (5.6, 6.6) times more likely to be chosen as healthy. Foods with better colours on saturated fat and salt were 7.3 (6.7, 8.0) and 7.1 (6.5, 7.8) times more likely to be chosen as healthy – significantly greater than for total fat (odds ratio 4.8 (4.4, 5.3)) and sugar (5.2 (4.7, 5.6)). Results were broadly similar for different genders and age groups.

**Conclusions:**

We found that participants were more concerned with avoiding reds than choosing greens, and that saturated fat and salt had a greater influence on decisions regarding healthiness than total fat and sugar. This could influence decisions about food reformulation and guidance on using nutrition labelling.

## Background

A healthy diet can protect against a number of non-communicable diseases including cardiovascular diseases and some cancers [1, 2]. The World Health Organization (WHO) suggests that nutrition labelling plays an important role in improving health by informing consumers of the nutritional quality of the foods they consume [3]. Research has shown that consumers who read nutrition labelling have a healthier diet [4] and that labels can help with weight loss [5]. However, some consumers have difficulty understanding traditional back-of-pack nutrient declarations [6] and it has been suggested that supplementing the back-of-pack nutrition information table with a front- of-pack (FOP) label may be more effective in encouraging consumers to choose healthier foods when shopping [7–9].

In the UK, a government-approved voluntary scheme for FOP nutrition labelling involves using colour-coded labels to indicate ‘low’, ‘medium’ or ‘high’ levels of total fat, saturated fat, total sugar and salt [10]. This system is often referred to as ‘traffic light labelling’, due to the red, amber and green colours used to represent low, medium and high amounts of each nutrient. Other countries that have introduced traffic light nutrition labelling schemes include Ecuador [11] and South Korea [12]. Sri Lanka have recently announced that they plan to introduce a voluntary traffic light scheme to label sugar content in beverages [13].

In the UK scheme, each colour and nutrient has its own thresholds. For example, to carry a green light for total fat a product must not contain more than 3 g of fat per 100 g for foods (1.5 g per 100 ml for drinks), and to carry a green light for sugar a food must not contain more than 5 g of sugar per 100 g (2.5 g per 100 ml for drinks). A range of front-of-pack label systems were studied by the UK Foods Standards Agency (FSA) from around 2006 to 2009 using a variety of methods to assess consumers’ understanding and acceptability of the labels. Traffic light labelling was identified as the most effective scheme and the FSA supported its use [14]. Since then all the major food retailers in the UK including the two largest (Tesco and Sainsbury’s), and some manufacturers including Nestle and PepsiCo have adopted the system. Choice experiments have shown that traffic light labelling is better than standard nutrient declarations at helping individuals choose healthier products [15] and that traffic light labels can increase the willingness to pay for low fat food products [16].

Increasing the prevalence and use of traffic light labelling could be a cost-effective obesity prevention strategy [17]. Traffic light labels have been shown to be an efficient way of promoting healthier food choices even under time constraints [18]. Unlike standard nutrient declarations traffic light labels are interpretive and provide a guide to important nutrients [19]. Research using mock products has found that participants were five times more likely to identify healthy products using traffic light labels than other FOP nutrition labelling systems such as those that show content of nutrients as a percentage of recommended intake [20]. However, traffic light labelling has also been met with criticism due to what can be deemed as a simplification of a healthy diet and its focus on ‘negative’ nutrients. It has been suggested that traffic light labels may ‘stigmatise’ certain foods that can otherwise be incorporated into a healthy diet [21].

Whilst there have been number of very comprehensive reviews on nutrition labelling and its effect on consumer perceptions and choice [6, 22–24] it remains unclear exactly how shoppers use the information presented in traffic light labels to make decisions about the healthiness of foods. For example they may place different emphasis on different nutrients [25], or give greater prominence to negative attributes (red lights) than positive attributes (green lights) [26] – an example of ‘negativity bias’ [27]. We conducted an online choice experiment to investigate how UK shoppers use the different features of the traffic light label to inform decisions about the healthiness of foods. The three key research questions for this study were: Which of the four nutrients has the most influence on decisions about the healthiness of foods? Do greens or reds have a greater influence on decisions about the healthiness of foods? Are there any differences in the influence of different features for different sexes and different age groups? Understanding how people combine the different information in a traffic light nutrition label is important for informing interventions that could increase their use, thereby increasing their relevance as a public health tool.

## Methods

**Fig. 1:**
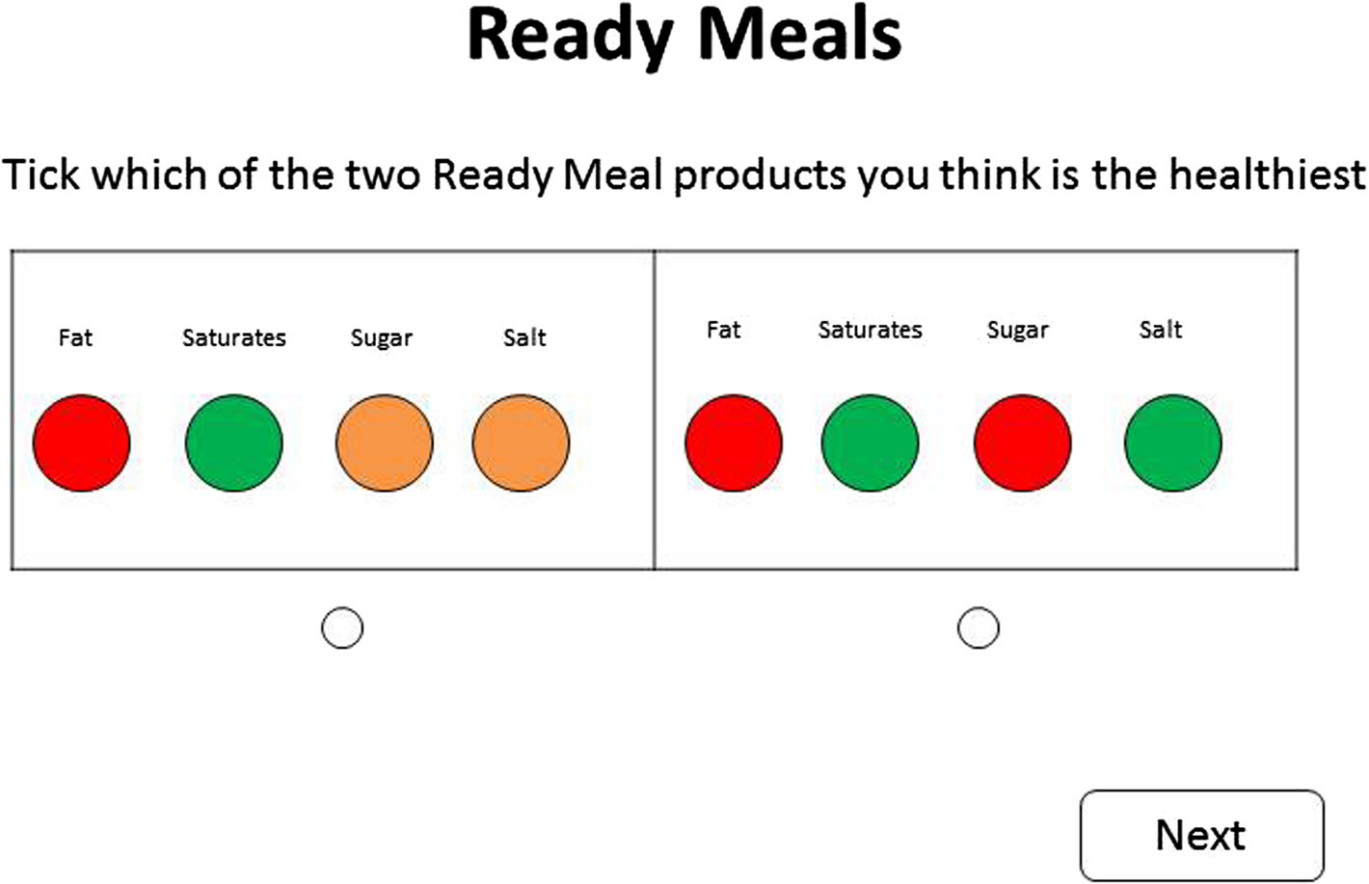
Example question from the online questionnaire

We designed an online questionnaire that asked participants to make a series of choices between two different traffic light labels, stripped of all context apart from the information about colours and nutrients. The participants were asked to choose which of the two traffic light labels represented the healthier food (in all cases, the foods were described as ‘ready meals’, a range of foods which the government recommends should have traffic light labels [28] and where traffic light labels are highly prevalent [29]). An example question taken from the questionnaire is shown in Fig. 1.

A set of 25 different traffic light labels was generated for the questionnaire, which were designed to cover the full range of nutritional quality of ready meals as identified in an audit of 373 ready meals in a single large supermarket in the UK conducted in 2012, and with reference to the New Zealand Nutritrack database of nutritional composition of packaged foods [30]. This set of 25 labels generated a total of 300 pairwise comparisons. Each participant was presented with a set of 20 pairwise comparisons randomly selected from the population of 300 comparisons and they were forced to decide which label was healthier, with no time constraints. After completion of the choice experiment, participants were asked descriptive questions on age, sex, ethnicity and socioeconomic status and nutrition literacy, which was measured by agreement with eight statements assessing the general health interest of the participants (e.g. ‘I always follow a healthy and balanced diet’) [31] and agreement with five statements assessing the subjective nutrition knowledge of participants (e.g. ‘I know a lot about healthy eating’) [32].

### Pilot stage

An early version of the study questionnaire was piloted with an opportunistic sample of 23 regular food shoppers living in Oxfordshire. The sample was targeted to recruit a broad range of age groups and an equal gender mix. The pilot sample was emailed a link to the questionnaire and was then contacted by a researcher who conducted a semi-structured interview about their experience of completing the questionnaire. The interview questions investigated functionality of the questionnaire and methods used by participants to inform their decisions. The pilot questionnaire included labels referring to three different food categories (with 20 comparisons in each category): ready meals, breakfast cereals and desserts. Analysis of questionnaire responses and interview replies indicated that participants did not use different techniques for different food categories and that participant engagement dipped after 20 comparisons, therefore only ready meals were retained for the questionnaire for the main study.

### Recruitment

Participants for the main study were recruited through the loyalty card list of a UK supermarket chain which comprises more than 1.1 million members. No incentive was provided for study participation. In order to be eligible participants had to be over the age of 18 and have used their loyalty card within the previous month. Emails were sent by the supermarket chain to a random sample of eligible shoppers and anonymised results were automatically transferred to the research team. We powered the analysis to detect an odds ratio of 2.0 (alpha = 0.05; beta = 0.8) for choosing a food as healthier if it had an improved colour on a single nutrient, adjusted for other nutrients, assuming a small correlation between the nutrient colours. Our power calculations suggested we needed approximately 1100 label comparisons to detect such an effect. Each participant provides 20 comparisons, so we aimed to have at least 55 participants for each sub-analysis. We aimed to conduct sub-analyses by gender and broad age group for which we expected a 5 % response rate and for only 25 % of respondents to be male, so we sent out 5,000 email invitations with a follow-up email 1 week after the initial invitation.

### Statistical analyses

We conducted multi-level logistic regression analyses where the unit of analysis was individual comparisons nested in study participants (i.e. individual participants could contribute up to 20 different comparisons). The outcome measure was whether or not the left-sided food was chosen as the healthier food; and the explanatory variables included (depending on the analysis) whether or not the traffic light colour for each nutrient (separately) is ‘better’ for the left hand food (green better than amber better than red), whether or not the left-sided food has more green lights, and whether or not the left-sided food has more red lights. We conducted chi-squared tests to assess whether the ‘better colour’ across comparisons were correlated between nutrients, and then both univariate and multiply adjusted logistic regression analyses were conducted. Random intercept models were calculated using MLwiN [33] and age and gender stratified models were calculated using Stata v S.E 11.2 [34]. All data for participants that did not provide answers to the age and gender questions were excluded from the age- and gender-stratified analyses.

Multi-level logistic models were also used to estimate whether the relative influence of green, amber and red lights was equivalent for the four nutrients on the traffic light. Here, for each nutrient (in mutually adjusted models) the dependent variables were dummy variables identifying differences in the compared foods where green was compared with amber, and also when amber was compared with red.

Finally, we conducted an analysis to estimate the relative ‘weight’ participants applyed to the green, amber and red traffic lights, in order to assess whether the amber light was considered closer to green or to red in evaluations of healthiness. To do this, we derived a ‘healthiness’ scale for all traffic light labels which combined the information on traffic light colours linearly with equal weight to each nutrient. The healthiness scale ranged between 0 for the least healthy food and 1 for the most healthy food. As the least healthy label consists of four red colours, this means that the weight for a red light is 0.00. As the most healthy label consists of four green colours, this means that the weight for a green light is 0.25. The analysis was designed to estimate the weight for the amber light that best fitted the results from the questionnaire. We created 25 different scales, where the weight for the amber light ranged from 0.00 to 0.25. For each scale, we performed a logistic regression with the outcome variable being whether or not the scale scored the left-sided food as healthier (Yes/No), and the exposure variable being whether or not the participant rated the left-sided food as healthier (Yes/No). We then compared the pseudo-R2 statistic for goodness of fit for each of the 25 healthiness scales, to see which weight for the amber light had the greatest fit with the questionnaire data.

### Ethics, consent and permissions

This study received ethical clearance from the University of Oxford Central University Research Ethics Committee (reference number SSD/CUREC1/13-060). The anonymised dataset is available upon request from the corresponding author.

## Results

There was a 3.8 % response rate to the initial or follow up invitation email, with 200 participants starting the questionnaire. Of these, 183 participants completed the demographic questions and some of the pairwise comparisons. This was slightly fewer participants than aimed for, but still achieved the power required for the gender-stratified sub-analyses as we recruited a greater percentage of male participants than we anticipated (38 % compared to an anticipated 25 %). The results below are restricted to this set of 183 participants. In total, 3321 comparisons were made with an average of 18.1 per participant. All 20 comparisons were completed by 165 of the participants.

Table 1 reports on the demographics of the participants. Of those who responded to the question, 62 % were female. More than 80 % were over the age of 45, and more than half (54 %) were educated to degree level or higher (in comparison, only 27 % of English adults aged 16 and over were educated to equivalent of a certificate of higher education in the 2011 census [35]). The sample overwhelmingly (97.5 %) consisted of people identifying with the ‘white/white British’ ethnic group. To achieve reasonably similar numbers of participants in each group, the age-stratified analyses were grouped into those aged 18–55 and 56+. Both general health interest and subjective nutrition knowledge were normally distributed within the sample. There was a small but significant difference in general health interest with greater interest in the older age group (*p* = 0.007), but there was no significant difference by sex. Subjective nutrition knowledge was significantly greater in women (*p* = 0.002) (but the magnitude of the difference was small), but there was no significant difference by age group.

**Table 1:** Descriptive statistics for 183 participants

	*Number (%)*		*Number (%)*
*Gender*		*Age when finished education*	
Female	102 (61.8)	Not yet finished	0 (0.0)
Male	63 (38.2)	Never went to school	0 (0.0)
Missing	18	14 or under	3 (1.8)
Age		16 years	44 (26.8)
18 to 25	3 (1.8)	18 years	29 (17.7)
26 to 35	8 (4.9)	19 years or over	88 (53.7)
36 to 45	21 (12.7)	Missing	19
46 to 55	38 (23.0)	*Highest qualification*	
56 to 65	57 (34.6)	Degree or equivalent	88 (53.7)
66 to 75	31 (18.8)	A level	15 (9.2)
76+	7 (4.2)	O level/GCSE	19 (11.6)
Missing	18	School certificate	9 (5.5)
Ethnicity		NVQ	6 (3.7)
White/white British	158 (97.5)	Trade apprenticeship	6 (3.7)
Asian/Asian British	2 (1.2)	Clerical/commercial qual	3 (1.8)
Black/African/Black British	0 (0.0)	Other	18 (11.0)
Mixed/multiple groups	0 (0.0)	Missing	19
Other ethnic group	2 (1.2)	*Employment*	
Missing	21	Full time student	1 (0.6)
		Employed	79 (47.9)
		Employment training	0 (0.0)
		Unemployed	6 (3.6)
		Unable to work	2 (1.2)
		Retired	60 (36.4)
		Permanent carer	10 (6.1)
		Other	7 (4.2)
		Missing	18

Chi-squared tests revealed strong correlations between the nutrients with regard to ‘better colours’. Total fat was positively correlated (*p* < 0.01) with saturated fat, sugar and salt, meaning that a food in a comparison with a better colour for total fat was also more likely to have a better colour for the other nutrients. However, salt was negatively correlated with both satuated fat and sugar (*p* < 0.01). Table 2 shows the results of the logistic regression models for the whole sample. Large differences between the odds ratios from the univariate and multivariate models (particularly for salt) are explained by the strong correlations between nutrients described above. After adjustment for colours on the other nutrients, saturated fat and salt emerged as the nutrients that had the biggest influence on decisions about healthiness. A better colour on the saturated fat nutrient increased the odds of selection as ‘healthier’ by 7.3 times (95 % confidence intervals: 6.7, 8.0) compared to only 4.8 times (4.4, 5.3) increased odds if there were a better colour on the total fat nutrient. The food with more greens in the pairwise comparison was 6.1 times (5.6, 6.6) more likely to be selected as healthy. However, the green effect was outweighed by the red effect. The odds ratio for selecting a food as more healthy if it had more reds was 0.09 (0.08, 0.10), which implies that a food was 11.4 times (10.3, 12.5) less likely to be selected as healthier if it had more reds.

**Table 2:** Odds ratio for selecting a food as healthier if it has a) more favourable colour for a nutrient; b) more greens or more reds

	*Univariate odds ratio (95 % confidence intervals)*	*Multivariate odds ratio (95 % confidence intervals)*
*a) Favourable colours on individual nutrients* ^*1*^		
Total fat	5.31 (5.01, 5.62)	4.83 (4.41, 5.29)^2^
Saturated fat	7.05 (6.61, 7.52)	7.31 (6.66, 8.02)^2^
Total sugar	3.94 (3.74, 4.16)	5.16 (4.72, 5.64)^2^
Salt	2.05 (1.96, 2.14)	7.11 (6.46, 7.81)^2^
*b) More greens or more reds*		
More greens	7.84 (7.36, 8.35)	6.09 (5.61, 6.61)^3^
More reds	0.07 (0.07, 0.08)	0.09 (0.08, 0.10)^3^

^1^For each nutrient, ‘favourable colour’ means a comparision of either ‘green: amber’, ‘green: red’ or ‘amber: red’, with equal weight placed on all such comparisons. ^2^Results are adjusted for superior colours on each of the other nutrients. ^3^Results are mutually adjusted for ‘more greens’ and ‘more reds’

Table 3 shows results stratified by sex and age. The results for men and women are very similar, with overlapping confidence intervals in all cases. The largest gender difference in the results was for saturated fat, which was considered more important by woman who were 9.0 times (7.0, 11.6) as likely to categorise a food as healthier if it had a favourable light for saturated fat, compared to only 5.8 times (4.4, 7.7) as likely for men. The results stratified by age revealed that younger and older age groups followed similar patterns across nutrients and colours but in all cases the odds ratios for the older age groups were lower than for the younger group. For example, those aged 55 and under were 17.3 times (12.1, 24.6) more likely to select a food as *less* healthy if it had more reds, compared to only 9.1 times (7.2, 11.5) for those aged 56 and over. The similar pattern but with reduced magnitude for elder participants is due to the greater variance in responses for the older age group.

**Table 3:** Multivariate results for favourable colours on nutrients and more greens or more reds stratified a) by gender and b) by age group

	*Multivariate odds ratio (95 % confidence intervals)*	
*a) Stratification by gender*		
	WOMEN	MEN
*Favourable colours on individual nutrients*		
Total fat	4.40 (3.43, 5.65)	4.65 (3.36, 6.43)
Saturated fat	9.02 (7.01, 11.59)	5.78 (4.36, 7.66)
Total sugar	4.71 (3.67, 6.04)	5.01 (3.69, 6.82)
Salt	7.16 (5.61, 9.13)	7.23 (5.33, 9.82)
*More greens or more reds*		
More greens	6.71 (5.37, 8.39)	5.29 (4.08, 6.86)
More reds	0.08 (0.06, 0.10)	0.10 (0.07, 0.13)
*b) Stratification by age*		
	18 to 55	56+
*Favourable colours on individual nutrients*		
Total fat	5.33 (3.79, 7.51)	3.97 (3.11, 5.07)
Saturated fat	11.95 (8.39, 17.02)	6.04 (4.83, 7.55)
Total sugar	7.10 (5.04, 9.99)	3.86 (3.05, 4.90)
Salt	9.49 (6.70, 13.43)	6.20 (4.94, 7.79)
*More greens or more reds*		
More greens	8.49 (6.27, 11.49)	5.10 (4.14, 6.27)
More reds	0.06 (0.04, 0.08)	0.11 (0.09, 0.14)

The results of multi-level logistic regressions comparing the effect of green, amber and red colours on each nutrient separately showed that, for each nutrient, the odds ratio for choosing a food as healthier if it had an amber compared to a red light was about twice as high than if it had a green light compared to an amber light (confirming the pattern demonstrated in Table 2). There was little evidence to suggest that participants were treating colours with different weights for different nutrients.

**Fig. 2:**
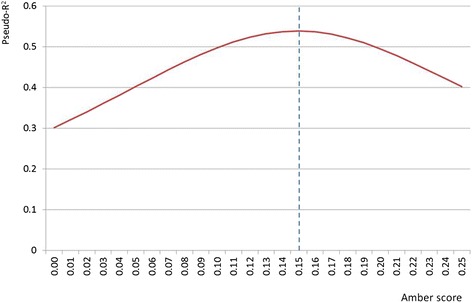
Pseudo-R^2^ for fit of logistic regression of likelihood of selecting a food as healthier if it has a healthier score (RED = 0.00; GREEN = 0.25; AMBER ALLOWED TO VARY BETWEEN 0.00 AND 0.25)

Figure 2 shows the results of the analysis comparing the weight provided to the amber light by the participants, and shows that the best fit with the responses from the participants is achieved when the amber light is given a weight of 0.15 (in comparison to 0.00 for red and 0.25 for green), implying that the participants considered an amber light to be closer to a green light than to a red light. This is also implied by the results of the logistic regression analyses which showed that participants were more influenced by the red lights than by the green lights. At the optimal weighting for the amber scores, the pseudo-R^2^ for a regression between ‘healthiness’ scores and participant responses was 0.54, implying that 54 % of the participant responses are predicted by a healthiness scale where red lights score 0.00, amber lights score 0.15 and green lights score 0.25.

## Discussion

Our sample of 183 UK supermarket shoppers showed that, when front-of-pack colour-coded nutrition labels are used to decide between the healthiness of foods, greater emphasis is placed on saturated fat and salt than total fat and sugar, and avoidance of red lights is more important than selection of green lights. Sub-analyses stratified by age and gender and qualitative findings from the pilot stage suggest that these outcomes are similar across different population groups, with one possible exception that needs further research to confirm: men do not seem to give as great an emphasis to saturated fat as women do. It should be noted that real-life food purchasing decisions have myriad influences (e.g. price, taste), but the results presented here suggest that, all else being equal, reformulation of products to move from from red to amber lights is likely to have a greater impact on the consumer than a movefrom amber to green lights. It is unclear what the public health implications of such reformulation would be and this should be a subject for future research. The results here have provided an insight into how UK consumers might use traffic light labelling to guide decisions about the healthiness of foods, which has implications for both the food industry and public health awareness campaigns such as Change4Life [36] or guidance on using nutrition labelling on foods.

The increased influence of saturated fat and salt suggests that this sample was more concerned about the links between these nutrients and health outcomes than for total fat and sugar. This may reflect the make up of the sample, who may have different health concerns to the general population. It may also reflect the well-publicised salt campaign conducted by the Food Standards Agency in the mid 2000s [37], and results here may not be generalizable to other countries with different public health campaigns. The data collection was conducted in May 2014, when the evidence relating saturated fat and health outcomes was being debated [38, 39] and the evidence base relating sugar consumption with adverse health outcomes was being reinforced [40, 41]. Our results suggest that at the time of this survey the debate over sugar and satuarated fat that had been playing out in the nutrition journals and the popular media had not changed the opinion of the participants over the relative contributions of these nutrients to poor health.

Two previous studies have investigated the relative influences of different elements of the traffic light labelling system, both of which used choice experiments. Balcombe et al. [42] applied traffic light labels to baskets of foods at different prices and asked 477 people from the UK to choose which they would buy. Their results concurred closely with ours, in that the willingness to pay for a shift from red to amber lights was much higher than that for a shift from amber to green lights, and that of the four nutrients under investigation, saturated fat and salt were more influential than total fat and sugar. They presented their traffic light label in a different order to the standard UK label, with salt first, then sugar, fat and finally saturates. It was therefore not clear whether the greater emphasis on salt and saturated fat may have been due to order effects, with greater emphasis being placed on nutrients that appear on the boundaries of the label. Our study used the standard ordering of total fat, saturated fat, sugar and salt, yet replicated the results that saturated fat and salt are the most influential nutrients. The sample recruited by Balcombe et al. was on average of lower income than the general UK population, suggesting a lower socioeconomic status – this is in contrast to our sample which is more educated than the norm.

Hieke and Wilczynski (2011) [25] conducted an online choice experiment with 2002 German students and asked them to choose between sets of three different traffic light labels for yoghurts. They also reported that the shift between red and amber was more influential than the shift between amber and green. However, they found that the two most influential nutrients were total fat and sugar, which contrasts with our findings and those of Balcombe et al. [42]. As with Balcombe et al., Hieke and Wilczynski did not use the standard UK order for nutrients, instead presenting traffic light labels with total fats first, sugar second, saturated fat third and sodium last. There could be a number of reasons for the contrasting results. First, the age profile of the Hieke and Wiczynski study was considerably younger than the other two studies, with 70 % of the participants under the age of 25. Second, the choice of ‘yoghurt’ as the standard food product for the choice sets may have influenced participants towards fat and sugar as influential nutrients. Third, the results may reflect cultural differences between Germany and the UK.

Our study is based on a sample of regular UK supermarket shoppers from a large supermarket chain that uses traffic light labelling on its own brand products. The participants are therefore drawn from the population who regularly encounter traffic light labelling in real-life shopping scenarios. The design of the study allowed us to concentrate on specific details of the labels (only on colours and nutrients), which allowed us to identify how these elements are used to make decisions regarding the healthiness of foods in isolation from other competing influences such as price, previous purchase, calorie labelling, and health and nutrition claims. The novel statistical techniques used here allowed us to develop a ‘healthiness’ scale, which is a data-driven score that can be applied to any food carrying traffic light labels. It should be noted that this healthiness scale may not reflect the true healthiness of the food because it is derived from the levels of just four nutrients and also on how consumers place weight on those levels. For example: diet cola, bottled water, pasta and broccoli would all have four greens and therefore have a healthiness score of 1.00 under this scale but there are clear, health-related, nutrient differences in these four foods. However such a healthiness scale will be useful for researchers investigating traffic light labelling [29] and could be used by health practitioners designing tools to help people use traffic light labels to make healthier food choices.

A strength of the study was that the multilevel deisgn of the analyses allowed us to control for differences in techniques used by different participants to combine the information on the labels. An important limitation of the study was that the study sample was not representative of the UK adult population. For example, in comparison to data collected from the 2011 census [35], our sample was more likely to be female and older, more highly-educated and less ethnically diverse. Also, whilst the traffic light profiles used in the survey were drawn from a realistic range of labels that appear on ready meals in the UK, the profiles used were not necessarily representative of all labels in the UK. For this reason, it is preferable to use the results of the adjusted analyses, which account for confounding due to potentially spurious nutritional correlations in the nutrition label comparisons. To derive the healthiness scale we assumed that the pairwise comparison was the unit of analysis (ignoring the multilevel structure used in the other analyses). That is we assumed that each pairwise comparison was conducted independently, which was not the case as each participant contributed up to 20 pairwise comparisons. If we assume that each participant used the same strategy to decide which food was healthier then this limitation would be reduced, and evidence from the pilot stage and the subgroup analyses suggests that similar strategies and techniques were used. However, it is not possible to assess whether this was always the case.

Future research in this area should focus on how FOP labelling is used in broader contexts than those studied here. For example, research could be expanded to consider other elements of the FOP nutrition label, including calorie content, written signifiers of ‘high’, ‘medium’ and ‘low’, and percentage contribution to Reference Intakes for nutrients – all features that commonly appear on FOP nutrition labels [10]. Further, the influence of the FOP label in its entirety could be considered alongside other elements of food labelling, such as health and nutrition claims, and work could be conducted in real shopping situations to investigate the influence of FOP nutrition labels. Such insight is needed to understand how FOP nutrition labels influence purchasing behaviour and how interventions can be developed to increase their influence.

## Conclusion

Our sample of UK supermarket shoppers were more concerned with avoiding reds than choosing greens. Saturated fat and salt had a greater influence on their decisions regarding healthiness than total fat and sugar. This could influence decisions about food reformulation and guidance on using front-of-pack nutrition labelling.

## References

[1] World Health Organization (WHO) (2007). Prevention of cardiovascular disease.

[2] American Institute of Cancer Research (AICR) / World Cancer Research Fund (WCRF) (2007). Food, nutrition, physical activity, and the prevention of cancer: a global perspective.

[3] World Health Organization (WHO) (2004). Global strategy on diet and physical activity.

[4] Kreuter MW, Brennan LK, Scharff DP, Lukwago SN (1997). Do nutrition label readers eat healthier diets? Behavioral correlates of adults’ use of food labels. Am J Prev Med.

[5] Laz T, Rahman M, Berenson A (2014). Association of frequent use of food labels with weight loss behaviors among low-income reproductive-age women. J Am Coll Nutr.

[6] Cowburn G, Stockley L (2005). Consumer understanding and use of nutrition labelling: a systematic review. Public Health Nutr.

[7] Geiger C, Wyse B, Parent C, Hansen R (1991). Review of nutrition labelling formats. J Am Diet Assoc.

[8] Scott V, Worsley A (1994). Ticks, claims, tables and food groups: a comparison for nutrition labelling. Health Promot Int.

[9] Institute of Medicine (2011). Examination of front-of-package nutrition rating systems and symbols: promoting healthier choices.

[10] Department of Health (2013). Final design of consistent nutritional labelling system given green light.

[11] World Cancer Research Fund. Nutrition labels. http://www.wcrf.org/int/policy/nourishing-framework/nutrition-labels Accessed 1st July 2015.

[12] European Food Information Council (2015). Global update on nutrition labelling.

[13] The Island. Health ministry takes first step to combat excess use of sugar by people. http://www.island.lk/index.php?page_cat=article-details&page=article-details&code_title=127431 Accessed 1^st^ July 2015.

[14] Malam S, Clegg S, Kirwan S, McGinigal S (2009). BMRB Social Research. Comprehension and use of UK nutrition signpost labelling schemes. A report for the FSA.

[15] Helfer P, Shultz T (2014). The effects of nutrition labelling on consumer food choice: a psychological experiment and computational model. Ann N Y Acad Sci.

[16] Drescher L, Roosen J, Marette S (2014). The effects of traffic light labels and involvement on consumer choices for food and financial products. Int J Consum Stud.

[17] Sacks G, Veerman J, Moodie M, Swinburn B (2011). ‘Traffic-light’ nutrition labelling and ‘junk-food’ tax: a modelled comparison of cost-effectiveness for obesity prevention. Int J Obes.

[18] van Herpen E, Trijp HC (2011). Front-of-pack nutrition labels. Their effect on attention and choices when consumers have varying goals and time constraints. Appetite.

[19] Jones G, Richardson M (2007). An objective examination of consumer perception of nutrition information based on healthiness ratings and eye movements. Public Health Nutr.

[20] Kelly B, Hughes C, Chapman K, Louie J, Dixon H, Crawford J (2009). Consumer testing of the acceptability and effectiveness of front-of-pack food labelling systems for the Australian grocery market. Health Promot Int.

[21] Holdsworth M, Delpeuch F, Kameli Y, Lobstein T, Millstone E (2010). The acceptability to stakeholders of mandatory nutritional labelling in France and the UK – findings from the PorGrow project. J Hum Nutr Diet.

[22] Campos S, Doxey J, Hammond D (2011). Nutrition labels on pre-packaged foods: a systematic review. J Public Health Nutr.

[23] Grunert K, Wills J (2007). A review of European research on consumer response to nutrition information on food labels. J Public Health.

[24] Kroonenberg-Vyth E (2012). Evaluation of a front-of-pack nutrition label. Effects on consumer behavior, product development and public health.

[25] Hieke S, Wilczynski P (2012). Colour Me In--an empirical study on consumer responses to the traffic light signposting system in nutrition labelling. Public Health Nutr.

[26] Grunert KG, Wills JM, Fernández-Celemín L (2010). Nutrition knowledge, and use and understanding of nutrition information on food labels among consumers in the UK. Appetite.

[27] Rozin P, Royzman E (2001). Negativity bias, negativity dominance, and contagion. Pers Soc Psychol Rev.

[28] Department of Health (2013). Guide to creating a front of pack (FoP) nutrition label for pre-packed products sold through retail outlets.

[29] Scarborough P, Hodgkins C, Raats MM, Harrington R, Cowburn G, Dean M, et al. Protocol for a pilot randomised controlled trial of an intervention to increase the use of traffic light food labelling in UK shoppers (the FLICC trial). BMC Pilot and Feasibility Studies. 2015;1:21. 10.1186/s40814-015-0015-1 PMC5153808 27965800

[30] Rosentreter S, Eyles H, Ni Mhurchu C (2013). Traffic lights and health claims: a comparative analysis of the nutrient profile of packaged foods available for sale in New Zealand supermarkets. Aust N Z J Public Health.

[31] Roininen K, Lähteenmäki L, Tuorila H (1999). Quantification of consumer attitudes to health and hedonistic characteristics of foods. Appetite.

[32] Flynn L, Goldsmith R (1999). A short, reliable measure of subjective knowledge. J Bus Res.

[33] Rasbash J, Charlton C, Browne WJ, Healy M, Cameron B (2009). MLwiN Version 2.1. Centre for multilevel modelling.

[34] StataCorp (2009). STATA version 11.

[35] Office for National Statistics (2011). Census: aggregate data (England and Wales). UK data service census support.

[36] National Health Service. Change4Life. http://www.nhs.uk/change4life/Pages/change-for-life.aspx Accessed November 2015.

[37] Shankar B, Brambila-Marcias J, Traill B, Mazzocchi M, Capacci S (2013). An evaluation of the UK Food Standards Agency’s salt campaign. Health Econ.

[38] Siri-Tarino PW, Sun Q, Hu FB, Krauss RM (2010). Meta-analysis of prospective cohort studies evaluating the association of saturated fat with cardiovascular disease. Am J Clin Nutr.

[39] Scarborough P, Rayner M, van Dis I, Norum K (2010). Meta-analysis of effect of saturated fat intake on cardiovascular disease: over adjustment obscures true associations. Am J Clin Nutr.

[40] Ebbeling CB, Feldman HA, Chomitz VR, Antonelli TA, Gortmaker SL, Osganian SK (2012). A randomized trial of sugar-sweetened beverages and adolescent body weight. N Engl J Med.

[41] Malik VS, Popkin BM, Bray GA, Despres JP, Hu FB (2010). Sugar-sweetened beverages, obesity, type 2 diabetes mellitus, and cardiovascular disease risk. Circulation.

[42] Balcombe K, Fraser I, Di Falco S (2010). Traffic lights and food choice: a choice experiment examining the relationship between nutritional food labels and price. Food Policy.

